# Label-Retaining Cells in the Adult Murine Salivary Glands Possess Characteristics of Adult Progenitor Cells

**DOI:** 10.1371/journal.pone.0107893

**Published:** 2014-09-19

**Authors:** Alejandro M. Chibly, Lauren Querin, Zoey Harris, Kirsten H. Limesand

**Affiliations:** 1 The University of Arizona, Cancer Biology Graduate Program, Tucson, Arizona, United States of America; 2 The University of Arizona, Department of Nutritional Sciences, Tucson, Arizona, United States of America; National Institute of Dental and Craniofacial Research, United States of America

## Abstract

Radiotherapy is the primary treatment for patients with head and neck cancer, which account for roughly 500,000 annual cases worldwide. Dysfunction of the salivary glands and associated conditions like xerostomia and dysphagia are often developed by these patients, greatly diminishing their life quality. Current preventative and palliative care fail to deliver an improvement in the quality of life, thus accentuating the need for regenerative therapies. In this study, a model of label retaining cells (LRCs) in murine salivary glands was developed, in which LRCs demonstrated proliferative potential and possessed markers of putative salivary progenitors. Mice were labeled with 5-Ethynyl-2′-deoxyuridine (EdU) at postnatal day 10 and chased for 8 weeks. Tissue sections from salivary glands obtained at the end of chase demonstrated co-localization between LRCs and the salivary progenitor markers keratin 5 and keratin 14, as well as *kit* mRNA, indicating that LRCs encompass a heterogeneous population of salivary progenitors. Proliferative potential of LRCs was demonstrated by a sphere assay, in which LRCs were found in primary and secondary spheres and they co-localized with the proliferation marker Ki67 throughout sphere formation. Surprisingly, LRCs were shown to be radio-resistant and evade apoptosis following radiation treatment. The clinical significance of these findings lie in the potential of this model to study the mechanisms that prevent salivary progenitors from maintaining homeostasis upon exposure to radiation, which will in turn facilitate the development of regenerative therapies for salivary gland dysfunction.

## Introduction

Radiotherapy is the primary treatment for the nearly 500,000 annual cases of head and neck cancer in the world [1]–[4]. Although the goal of radiotherapy is to target the tumor, secondary exposure occurs in surrounding tissues, such as salivary glands and oral mucosa [5], [6]. Some of the complications that arise from damage to these normal tissues include acute mucositis, recurrent pneumonia, esophageal dilation, saliva depletion, increased oral infections, and difficulty breathing and swallowing [1], [3], all of which can last several months or even permanently, contributing to a miserable quality of life.

The FDA-approved drug amifostine has been extensively used in trials as a preventative treatment to ameliorate the side effects that follow radiotherapy [7]–[9]. Although a reduction of xerostomia has been observed with amifostine, the number of side effects and serious conditions such as hypotension, Stevens-Johnsons syndrome, and hypersensitivity [10], [11], often influence patients’ compliance. Sialogogues are used as palliative care to stimulate saliva flow when partial salivary gland function is retained, but their efficacy highly diminishes with decay of salivary gland function [12], [13]. Similarly, saliva substitutes are utilized to maintain moisture in the mouth, and to help preserve oral health; however, the use of substitutes is only a replacement therapy and not a cure for xerostomia [14]. Because both preventive and palliative care fail to improve quality of life of patients undergoing radiation therapy, it is necessary to develop regeneration therapies that allow for restoration of salivary gland function.

Adult progenitor cells have been proposed to have significant roles in wound healing responses, tissue homeostasis, and regeneration [15]–[17]. A previous review has suggested that chronic dysfunction of the salivary glands is due to improper DNA repair in progenitor cells, thereby impairing the ability of salivary glands for self-repair [18]. A major problem in the field of adult salivary gland progenitors is that their identity is still somewhat elusive, due to the lack of known specific markers to delineate such populations [19]. For this reason, even though salivary gland progenitors have been studied in models of salivary gland development, based on molecular markers identified in other exocrine tissues [20]–[22], they are limited by the extent of progenitor-specificity of these markers in the adult gland, and the purity of these populations.

Early studies seeking to isolate progenitor cells of developing salivary glands relied on the expression of c-kit, Sca-1, Keratin 5 (K5), Ascl3, and Keratin 14 (K14) [21]–[25], which have rendered mixed and heterogeneous populations, some of which retain some regenerative potential [22], [26], [27] but do not seem to perfectly overlap with one another [21], [25], suggesting the existence of multiple progenitor cells in the salivary epithelium.

Studies by Lombaert et al. [22], [26] reported that c-kit+ cells derived from ductal structures of murine submandibular gland, have self-renewal capacity and can differentiate into both acinar and ductal cells *in vivo* and *in vitro*. In these studies, c-kit positive cells demonstrated to have the ability to partially restore function of damaged salivary glands after transplantation; however, it remains unclear whether endogenous c-kit+ cells are relevant in wound healing or regeneration of the parotid gland. Additionally, lineage tracing assays have demonstrated that Ascl3 marks progenitor cells in all 3 major salivary glands [25], and Keratin 5 is present in submandibular proximal progenitors confined to the basal layer of the ducts [21], [23]. Interestingly, Ascl3+ cells were described as a restricted population of progenitors, since they did not generate serous acinar cells and were not precursors to K5+ cells [25]. Similarly, K5+ and c-kit+ cells share some co-localization during salivary gland development [21], but both have been described as different progenitors [23], [26], [28]. Only recently, a study looked at cells that co-express c-kit and K14, which proved important for branching morphogenesis [26]. However, the role of these populations in homeostasis of the adult parotid and submandibular glands remains to be elucidated.

In order to circumvent the caveats of identifying progenitor cells based on the expression of molecular markers, some techniques have been developed such label retaining assays, which allow for cell sorting techniques and lineage tracing studies [29]–[31]. Label retaining cell (LRC) assays have contributed in the past to the identification of progenitors in liver [32], skin [33], sweat gland [34], [35], pancreas [36], intestine [37], [38], and other tissues [39]–[43]. In the adult salivary glands, LRCs have been found distributed in all parenchymal structures [44], supporting the idea that multiple progenitors coexist to maintain the complex structure of the adult salivary gland. The significance of these findings lies on the potential to develop new therapies to restore function of the damaged salivary glands, which will be greatly facilitated upon identification of the progenitor cells responsible for maintaining homeostasis and function of the adult salivary glands. Moreover, it is vital to understand the mechanisms by which radiation therapy corrupts the function of these progenitors so that more efficient targeted therapies can be developed.

In this study we designed a pulse and chase assay utilizing BrdU or EdU injections in a mouse model to detect label retaining cells in the salivary glands. Salivary gland LRCs were present in all parenchymal compartments of the gland, and co-localized with a diverse population of progenitors including c-kit+, K5+, and K14+ cells. Upon culturing *in vitro*, LRCs initiated sphere formation and differentiated into amylase secreting cells. Finally, we demonstrate that LRCs are maintained following radiation treatment, which makes them extremely attractive for the study and development of regeneration therapies for damaged salivary glands.

## Materials and Methods

### Mice and label retaining assay

All experiments were conducted in FVB mice, both male and female and repeated at least twice and in most cases 3–4 times. For all experiments, date of birth was considered ‘Day 0’ unless otherwise specified. At day 10, mice were given four intraperitoneal BrdU (5-Bromo-2′-deoxyuridine, Roche, Mannheim, Germany) injections at a dose of 3 mg per 100 g of body weight, or EdU (5-ethynyl-2′-deoxyuridine, Invitrogen, Carlsbad, CA) injections at a dose of 10 mg/100 g of body weight 12 hours apart. The chase period for the label retaining assay was 8 weeks, and at this time point the mice were anesthetized via an intraperitoneal injection with Avertin (240 mg/kg, Sigma, St Louis, MO) and euthanized by exsanguination for collection of the salivary glands. All mice were maintained and treated in accordance with protocols approved by the University of Arizona Institutional Animal Care and Use Committee (IACUC). A total of 5 animals (3 males and 2 females) were labeled with BrdU and 46 animals (24 males and 22 females) were labeled with EdU. Mice were distributed to different experiments as indicated in the following sections.

### Immunohistochemistry and immunofluorescence staining

**Figure 1:**
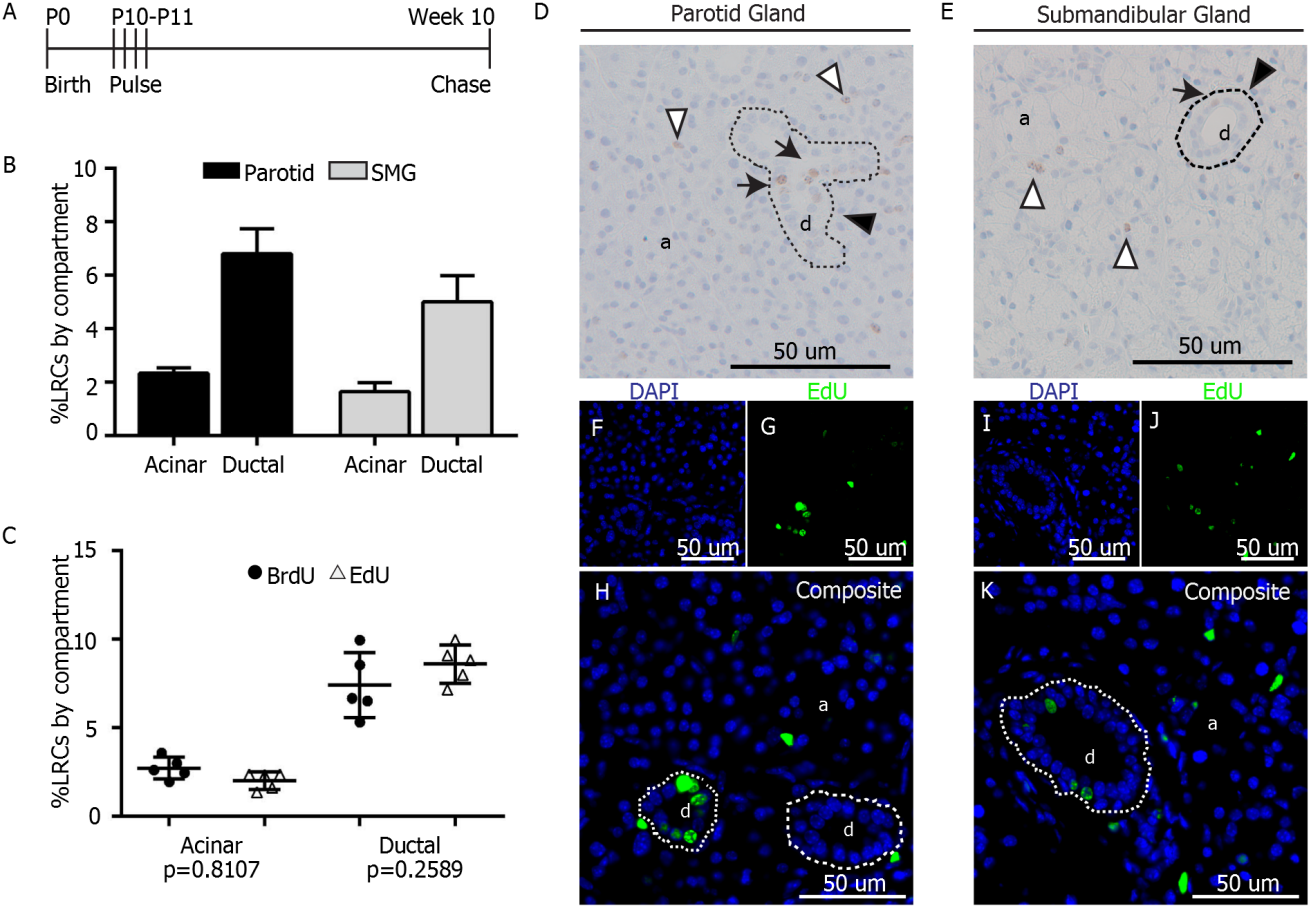
Label retaining assay in murine salivary glands. A) Label Retaining Assay. At 10 days after birth (P10), FVB mice were pulsed with 4 doses of EdU (100 mg/kg) or BrdU (30 mg/kg) 12 hours apart. Tissue was collected from 10-week old animals. B) LRCs from 3 mice were manually quantified per salivary gland compartment (acinar and ductal). Data are expressed as percentage from the total of cells in each individual compartment for both parotid and submandibular glands. C) Comparison of labeling efficiency between EdU and BrdU. Data are shown as percentage of LRCs per individual compartment. A 2-sided unpaired T-test was performed for analysis (n = 5 per group). D–E) Representative images of parotid and submandibular glands of BrdU-pulsed animals. LRCs in the acinar compartment (a) are shown with white arrowheads. LRCs in ductal compartment (d) are pointed with black arrows. Example of ductal compartment is delineated by dashed line and pointed with black arrowhead F–K) Representative fluorescent images of salivary glands from EdU-pulsed animals. EdU LRCs are shown in green and DAPI in blue.

Following dissection, the three major salivary glands were immediately fixed in 10% neutral buffered formalin (Sigma) for 24 hours, transferred to 70% ethanol, and embedded in paraffin. Sections of all major salivary glands were cut to 4 µm thickness and processed for standard staining with hematoxylin and eosin by the Histology Service Laboratory in the Department of Cell Biology and Anatomy at the University of Arizona. Slides were incubated at 37°C for 20 minutes and rehydrated in Histo-clear (National Diagnostics, Atlanta, GA), graded ethanol (100%–50%) and distilled water. Antigen retrieval was performed placing slides in 1 mM citric acid buffer (pH 6.8) and boiling in microwave twice for 5 minutes, plus additional 20 minutes in buffer without further microwaving. For BrdU immunohistochemistry, 0.3% Hydrogen peroxide was used to quench endogenous peroxidases for 15 minutes at room temperature prior to antigen retrieval. Slides were treated as instructed by the manufacturer (Vectastain Elite ABC kit, PK-6104, Vector Laboratories, Burlingame, CA). Primary Rat monoclonal antibody anti-BrdU (ab6326, Abcam, Cambridge, England) was used overnight at 4°C. Positive staining was developed using DAB (Biogenex. San Ramon, CA) for 5 minutes. Slides were counterstained with hematoxylin, dehydrated with graded ethanol washes (50%–100%) and mounted with Permount (Thermo Fisher Scientific, Pittsburg, PA). For immunofluorescence staining, slides were incubated in 0.02% Triton-x100 solution in 1X PBS for 15 minutes, followed by three 1X PBS washes of 5 minutes each prior to antigen retrieval. Slides were blocked in 300 µl of 0.5% NEN and incubated in primary antibody diluted in 1% BSA overnight at 4°C. After 3 consecutive washes with 1xPBS for 5 minutes each, secondary antibody was added for 1 hour at room temperature. Slides were rinsed with 1X PBS and washed with distilled water for 10 minutes. Finally, tissues were counterstained with DAPI (1 µg/mL) and mounted with a solution of 50% glycerol in 10 mM Tris-HCl (pH 8.0). Fluorescently stained slides were stored at 4°C for no longer than 5 days until imaging. Images were taken with a Leica DM5500 microscope (Leica Microsystems, Wetzlar, Germany) and 4 megapixel Pursuit camera (Diagnostic Instruments, Inc, Sterling Heights, MI) and ImagePro Software. BrdU and EdU-positive cells in parotid and submandibular sections were manually counted from a minimum of 5 fields of view (40X objective) per slide from three slides (three mice) per group. For evaluation of EdU and BrdU efficiency, 5 BrdU-labeled and 5 EdU-labeled mice (both male and female) were analyzed. Cells from acinar and ductal compartments were counted separately and statistical analysis was performed individually as described in the statistical analysis section. Ductal structures were identified based on morphological features (cellular structure enclosing a lumen, example marked with black arrowhead and dashed lines in Figure 1D–E), and were designated as ‘ductal compartment’. The rest of the glandular area was termed ‘acinar compartment’, which includes mostly acinar and myoepithelial cells. Each of these compartments also includes a minority of cells which are not traditional ductal or acinar cells. We used primary antibodies anti Keratin 14 (1∶400, PRB-155P, Covance, Princeton, NJ), Keratin 5 (1∶400, PRB-160P, Covance), Monoclonal Anti-Smooth Muscle alpha-Actin (1∶500, C6198, sigma), Ki67 (1∶200, 12202, Cell signaling Danvers, MA) and Amylase (1∶500, clone 1A4, Sigma). Each marker was stained for in tissues obtained from four 10-week old mice (2 males and 2 females), and six 10-day old mice (4 females and 2 males), and each experiment was repeated an average of 3 times.

### Flow cytometry and cell sorting

Immediately after dissection, glands from 2 female EdU labeled mice as well as 2 female non-labeled controls were minced in dispersion media containing 1 mg/ml of Collagenase (Sigma), 1 mg/ml of Hyaluronidase (Sigma) and 9 mM of CaCl_2_ in Modified Hanks Balanced Salt Solution (HBSS). Gland preparations were incubated at 37°C for 20 minutes with slight agitation. Cell suspension was pelleted by centrifugation at 100 g for 3 minutes and resuspended in 1 mL of 1 µM EGTA in HBSS. Cells were put in a rocker for 10 minutes at 37°C to allow for complete digestion. Further, cells were passed through a 20 µm nylon mesh to separate undigested tissue. Cells were centrifuged at 800 rpm at 4°C and washed with 1 mL of cold 1xPBS. Cells were pelleted again and resuspended in 500 µL of ice-cold PBS. Cells were fixed and stained for EdU following the manufacturer’s instructions (Click-iT EdU Flow Cytometry Assay Kit, Invitrogen). EdU+ cells were sorted using the FACSAria (BD Bioscienes, San Jose, CA) and recovered in RNAlater (QIAGEN, Venlo, Limburg) for PCR analysis. Additionally, a 10 µL aliquot of the recovered samples was placed in a microscope slide for imaging to confirm presence of EdU+ cells.

### Real-time PCR

Gene expression was evaluated from FACS-sorted EdU+ cells. Cells were sorted following FACS procedure described above and collected in RNAlater (QIAGEN) at 4°C. cDNA was obtained directly from cells using FastLane cell cDNA kit (QIAGEN). Real-time PCR was performed using 5 µL of cDNA from cells with the appropriate primers. SYBR Green (QIAGEN) was used for detection. All primers were obtained from Integrated DNA Technologies (Coralville, IA), and their sequences are as follows: amylase (Fwd: 5′-GCTCATCCTTATGGTTTCACACGG-3′, Rev: 5′-CCAGTCATTGCCACAAGTGCTGTC-3′), aquaporin 3 (aqp3, Fwd: 5′-CTGCCCGTGACTTTGGACCTC-3′, Rev: 5′-CGAAGACACCGATGGAACC-3′), Keratin 5 (krt5, Fwd: 5′-GAACAAAGGTGGAGGGAAGA-3′, Rev: 5′-TGCTGTCCCACCAAATCTTG-3′), Keratin 14 (krt14, Fwd: 5′-CTGGTGGGCAGTGAGAAAGT-3′, Rev: 5′-CCAGGATCTTGCTCTTCAGG-3′), kit (Fwd: 5′-TGGTTGTGGTTGTTGTTGTTGTTG-3′, Rev: 5′-GAAGGCTTGTTCCGAAGTGTAGAC-3′), and sox2 (Fwd: 5′-ATGGACAGCTACGCGCAC-3′, Rev: 5′-CGAGCCGTTCATGTAGGTCTG-3′).

### Fluorescent in-situ hybridization (FISH)

Formalin-Fixed Paraffin-Embedded slides from all major salivary glands were obtained from six 10-day old mice (4 females and 2 males) and four 10-week old EdU labeled mice (2 males and 2 females) as described in the previous sections. Slides were pretreated following the manufacturer’s instructions (RNAscope Fluorescent Multiplex, 320850, Advance Cell Diagnostics, Hayward, CA). Slides were incubated with a probe for mouse c-kit mRNA (*kit*) (NM_021099, Advance Cell Diagnostics, Hayward, CA) for 2 hours at 40°C. Following hybridization, slides were imaged with a Leica DM5500 microscope (Leica Microsystems, Wetzlar, Germany) and 4 megapixel Pursuit camera (Diagnostic Instruments, Inc, Sterling Heights, MI) using ImagePro Software.

### Sphere assay

A total of 8 EdU-labeled mice (4 males and 4 females) were used for sphere culture across 4 individual experiments. For each experiment, parotid and submandibular glands from 2 mice were collected at week 8 after pulse, and immediately placed in dispersion media containing a mixture of Collagenase and Hyaluronidase (5 mg/5 mL of modified Hanks solution per gland), 0.1% CaCl_2_, gentamycin (0.1 mg/mL, Life Technologies, Carlsbad, CA), and fungizone (5 µg/mL, Roche). Glands were minced until big clumps dissociated and then incubated at 37°C for 15 minutes with gentle agitation. Cell suspension was then passed through 40 µm mesh sterile filters into a new sterile conical tube and centrifuged at 4000 rpm for 10 minutes. Supernatant was discarded and pellet re-suspended in sphere culture media: DMEM/F12 containing streptomycin, penicillin, EGF (20 ng/ml, Fisher Scientifics, Waltham, MA), FGF2 (20 ng/ml, Sigma), Insulin (10 µg/ml, Invitrogen), N2 supplement (1X, Invitrogen), Dexamethasone (1 µM, Sigma) and glutamine (2.5 mM). Cells were plated at a density of 400,000 cells per well in ultra-low attachment plates (Corning, Corning, NY). For collection, plates were visualized under a bright-field microscope to confirm the presence of spheres. Cells were fixed directly in culture prior to collection by adding 1 volume of 10% buffered formalin for 30 minutes at room temperature. Fixing cells at this point helps prevent aggregation of spheres due to further centrifugation steps. After fixation cells were gently centrifuged for 10 minutes to discard the supernatant; cells were then permeabilized with 0.2% TritonX in PBS for 15 minutes at room temperature to initiate the staining procedure. EdU Staining was performed adapting the manufacturer’s instructions (Click-iT Plus EdU Alexa Fluor 488 Imaging Kit, Life Technologies, Grand Island, NY) to stain cells directly in suspension; cells were pelleted by centrifugation at 5000 rpm for 5 minutes and resuspended in EdU click-it cocktail for 30 min at room temperature covered from light. Target-specific staining of spheres was performed in suspension by adding primary antibody diluted 1∶200 in 2% BSA for 1 hour at room temperature, followed by incubation in secondary antibody anti-rabbit Alexa Fluor 594 (A-11037, Invitrogen) at 1∶500 dilution in 2% BSA. Between steps, a single wash with PBS was performed. All centrifugation steps after initial collection of spheres were performed at 5000 rpm for 5 minutes. Primary antibodies used were Ki67 (12202, Cell Signaling), and amylase (clone 1A4, Sigma). Amylase-stained spheres were also imaged with the Nikon C1si scanning confocal microscope at the Keck Imaging center at the University of Arizona. Staining of spheres was repeated 4 times for each marker at each collection time point.

### Secondary sphere assay

Primary spheres were grown as described above for 14 days. At this point spheres were mechanically disrupted by passing them through a 28 G needle several times and re-plated in fresh media in low-attachment plates. Observations on the day after plating showed that cells were present in small clusters of <5 cells, similar to a previously published report examining cellular clusters that form primary spheres [22]. Cells were cultured for an additional 7 days and collected to evaluate the presence of spheres.

### Statistical analysis

An unpaired two-sided T-Test for 2 samples with equal variances was utilized for comparison between BrdU and EdU (n = 5 for each group), and to evaluate statistical differences in % of EdU LRCs in irradiated samples (n = 7) versus untreated controls (n = 12 for parotid gland and n = 6 for submandibular gland). In all cases, data from the acinar compartment were analyzed separately from the ductal compartment. The number of animals utilized for analysis is also indicated under each experiment section as well as every figure legend.

### Radiation treatment

Mice were anesthetized via intraperitoneal injection with ketamine/xylazine (50 mg/kg/10 mg/ml) prior to radiation treatment. Radiation treatment consisted of a single dose of 5 Gy targeted to the head and neck region using a ^6^°Cobalt Teletherapy unit from Atomic Energy of Canada Ltd Theratron-80. The 5 Gy radiation dose was chosen based on our previous work demonstrating the dose caused elevated levels of p53 protein, activation of apoptosis and loss of salivary function [45]–[48]. The remaining body sections of the mice were protected with >6 mm thick lead to avoid systemic effects of radiation. Radiation dosage calculations and maintenance of the cobalt source are conducted by the Experimental Radiation Shared Service of the Arizona Cancer Center. A total of 10 EdU-labeled mice were treated with radiation, 7 of which (5 females and 2 males) were given the treatment at 4 weeks of age, and the remaining 3 (1 male and 2 females) were treated 24 hours prior to tissue collection at the end of the 8-week chase period. Analysis of % LRCs in irradiated animals was performed using 12 untreated controls (7 males and 5 females) for parotid gland and 6 untreated controls (4 males and 2 females) for submandibular gland, which were compared to specimens obtained from the 7 irradiated animals.

## Results

### Both acinar and ductal compartments of the salivary glands contain label retaining cells

Ten-day old FVB mice were given four EdU (10 mg/100 g of body weight) or BrdU injections (3 mg/100 gr of body weight), 12 hours apart to allow for binding of label to DNA of actively dividing cells (Fig. 1A). It was determined that 8 weeks was an optimal chase period (Fig. 1A) as the glands are fully developed at this point, and the number of LRCs in salivary glands represent only a minority of the total tissue, accounting for only about 4.25±0.25% of the parotid gland and 3.07±0.75% of the submandibular gland.

Chromogenic staining of BrdU was preferred initially to count the number of LRCs present in the salivary glands, since ductal structures were more easily visualized. However, multiplexing with different antibodies was not always possible with BrdU, and thus EdU was ideal for this application. LRCs were found throughout the gland in both acinar and ductal compartments (Fig. 1D, E). BrdU LRCs from 3 mice (2 males and 1 female) were counted manually in both acinar and ductal compartments to calculate the percentage of LRCs based on the total number of cells in each individual compartment. In parotid gland, 2.34% of the cells in the acinar compartment were LRCs, as well as 6.80% of the cells in the ductal compartment (Fig. 1B, D). In submandibular gland, 1.65% of the acinar area were LRCs, while 5.01% of the ductal compartment were LRCs (Fig. 1B, E). Because EdU staining was later used for multiplexing staining, the number of EdU LRCs (Fig. 1F–K) from 5 mice (3 males and 2 females) were also counted and compared to BrdU LRCs from 5 mice (3 males and 2 females) to confirm that the labeling efficiency of both compounds was comparable at the specified doses. The percentage of BrdU LRCs was not statistically different from the percentage of EdU LRCs (n = 5, p = 0.81 for acinar LRCs and n = 5, p = 0.25 for ductal LRCs) (Fig. 1C), which validates the use of both compounds interchangeably in our study.

### Long-lived LRCs of the salivary glands possess markers of putative progenitor cells

Progenitor cells have long believed to be localized to the ductal structures of the salivary glands [49], [50], but we found LRCs distributed in all parenchymal structures. As an initial screening test to determine whether salivary gland LRCs had the potential to be progenitor cells, EdU+ cells from EdU-labeled FVB mice were sorted at the end of the chase period by flow cytometry (Fig. S1A–K). Next we measured the expression of putative stemness-related genes in the salivary glands by real time PCR, such as *sox2*, *kit*, and *aqp3*, as well as the acinar differentiation marker *amylase*. Edu+ cells from parotid glands were enriched in markers *sox2*, *kit* and *aqp3*, and had lower *amylase* expression in comparison to EdU− cells (Fig. S1L).

**Figure 2:**
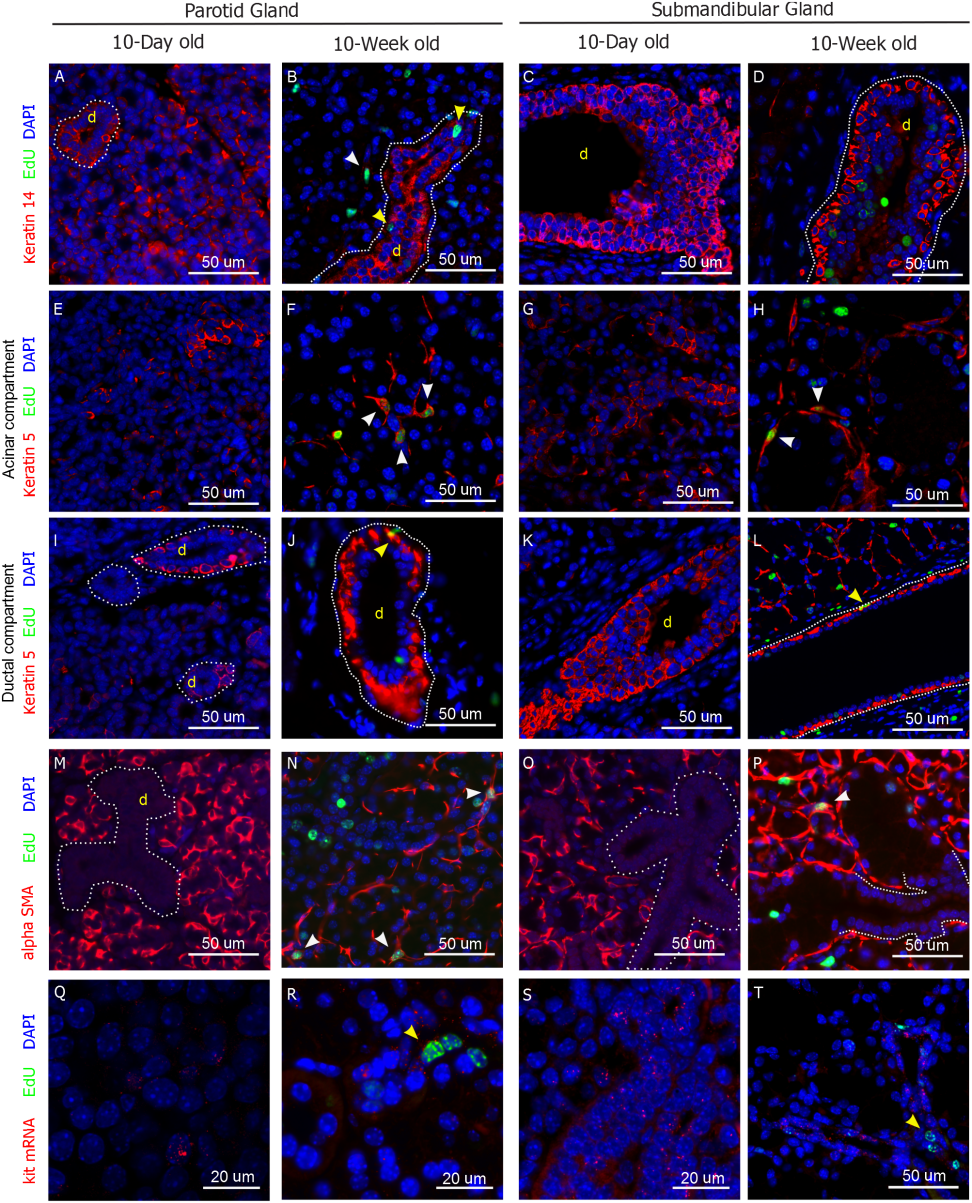
Molecular markers in salivary gland LRCs. Representative images of parotid and submandibular glands of 10-day old and 10-week old animals stained for Keratin 14 (A–D), Keratin 5 (E–L), Smooth Muscle alpha Actin (M–P). Q–T) Fluorescence in Situ Hybridization for kit mRNA. EdU LRCs are shown in green, DAPI in blue, and all other markers in red. White arrowheads point at co-localization of each marker with the LRCs in the acinar compartment. Yellow arrowheads point at co-localization of each marker with the LRCs in the ductal compartment. Full size images of every panel are shown in Figure S2.

In addition to c-kit, Keratin 5 (K5) and Keratin 14 (K14) have also been associated with salivary gland progenitors during development [23], [26], [27]; therefore we aimed to determine whether salivary gland LRCs expressed these markers *in vivo*. Immunofluorescence staining of tissue sections from 10-day old FVB mice was performed to confirm the presence of K5 and K14 in parotid and submandibular glands (Fig. 2, S2). Results were compared to immunofluorescence staining of adult tissue sections (10-week old mice) of EdU labeled animals.

In parotid glands of 10-day old mice, K14 was found in both acinar and ductal compartments (Fig. 2A, S2A), but it was almost exclusively found in ductal structures in 10-week old mice, where it co-localized with ductal LRCs (Fig. 2B, yellow arrowhead, S2B). A small group of LRCs in the acinar compartment co-localized with weakly stained K14+ cells (Fig. 2B, white arrowhead, S2B). In submandibular gland, K14 was found mostly in the excretory ducts of both 10-day old and 10-week old animals, with weak staining of cells in the acinar compartment (Fig. 2C–D, S2C–D). A small subset of submandibular LRCs co-localized with K14+ cells in the acinar compartment (not shown), but virtually no co-localization with ductal K14+ cells was observed (Fig. 2D, S2D). K5 was found in the basal layer of ducts, as well as distributed throughout the acinar compartment in 10-day old mice (Fig. 2E, I, S2E, I). In adults, basal localization of K5 was conserved (Fig. 2J, S2J), and K5+ cells in the acinar compartment had myoepithelial-like morphology (Fig. 2F, S2F). K5+ cells often co-localized (20.13%±2.47%, n = 5) with LRCs in the acinar compartment of parotid and submandibular glands (Fig. 2F, H, white arrowhead, S2F, H), while ∼1% of the ductal K5+ cells showed co-localization with ductal LRCs (Fig. 2J, L, yellow arrowhead, S2J, L). Staining with anti-Smooth Muscle alpha Actin (SMA) showed the presence of myoepithelial cells in both glands at 10 days of age (Fig. 2M, O, S2M, O), and demonstrated the myoepithelial nature of a number of LRCs in the acinar compartment of both glands in 10-week old animals (Fig. 2N, P, S2N, P). Fluorescence In-Situ Hybridization (FISH) was used for detection of *kit* mRNA in tissue sections. In parotid gland, *kit* RNA was found distributed throughout the gland in 10-day old animals (Fig. 2Q, S2Q), while it was expressed mostly in small ducts of the adult gland (Fig. 2R, S2R). In submandibular gland *kit* was mainly found in small ducts of both developing and adult glands (Fig. 2S–T, S2S–T) and weakly distributed to the rest of the tissue. LRCs often showed *kit* RNA expression in small ducts and acinar compartment cells of both parotid and submandibular glands (Fig. 2R, T, S2R, T), while no co-localization was observed in major ductal structures. These results confirmed the observations from the initial screening, which indicated that EdU+ from adult mice cells were enriched with c-kit (Fig. S1L).

Combined, these findings suggest that salivary gland LRCs are long-lived cells that are present in the salivary glands at postnatal day 10, and conserve their molecular signatures, such as the expression of K5, K14 and c-kit, as well as their localization. Additionally, co-localization of the LRCs with K5, K14, SMA and c-kit in multiple gland structures strongly suggests that LRCs are a heterogeneous population of putative progenitor cells.

### Salivary gland LRCs have proliferative potential

Although co-localization of LRCs with c-kit, K14 and K5 was evident, it is unknown whether these markers are specific for progenitor cells in the salivary glands, and despite their role during development, their contribution to the pool of adult progenitor cells is unclear. Moreover, although label retaining cells are long-lived cells that endure long periods of time in a state of quiescence, they are not necessarily progenitor cells [30], [31]. The sphere culture assay has been largely used as a tool to identify stem cells based on their capacity to self-renew and differentiate *in vitro* [51]. Therefore, to confirm that LRCs are progenitor cells, we sought to determine the capacity of salivary gland LRCs to form spheres *in vitro* under low-attachment conditions. Since LRCs co-localized with putative progenitor markers *in vivo*, we hypothesized that LRCs have the potential to expand and differentiate *in vitro* to form spheres.

**Figure 3:**
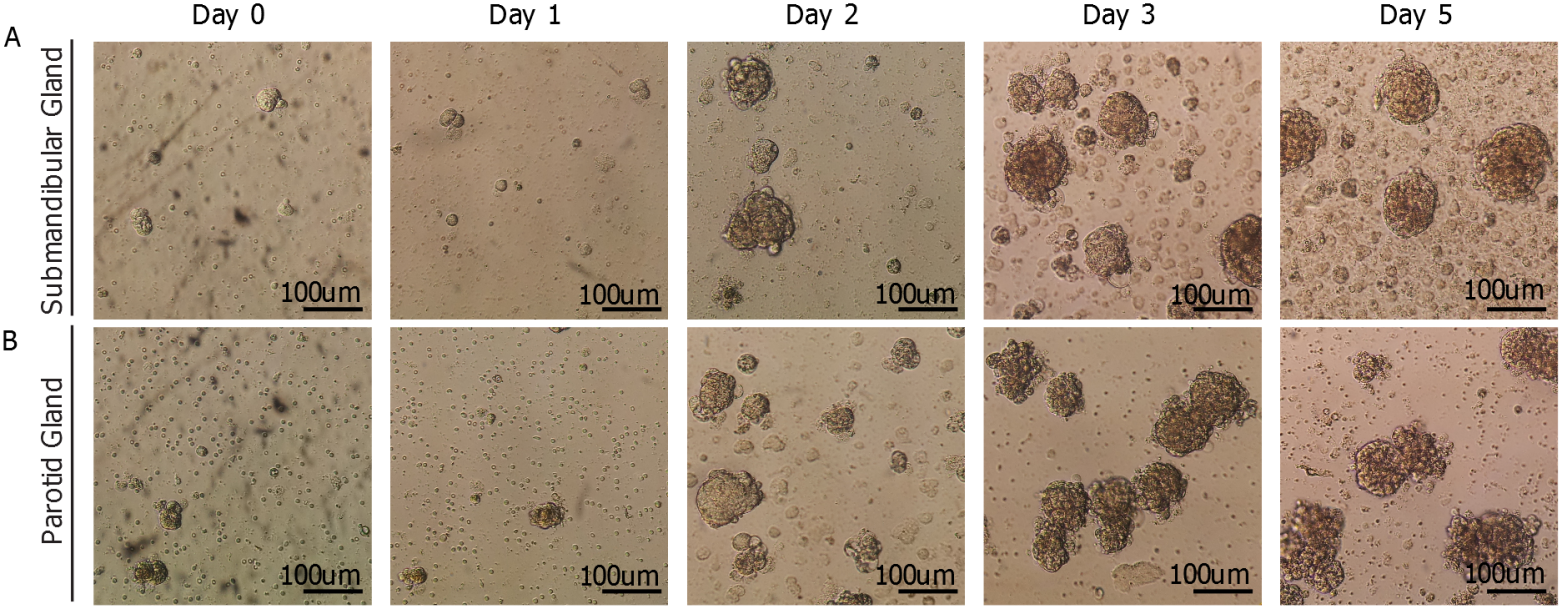
Sphere Assay with murine salivary glands. Representative microscope images of spheres grown from submandibular (A) and Parotid (B) glands from 10-week old mice.

**Figure 4:**
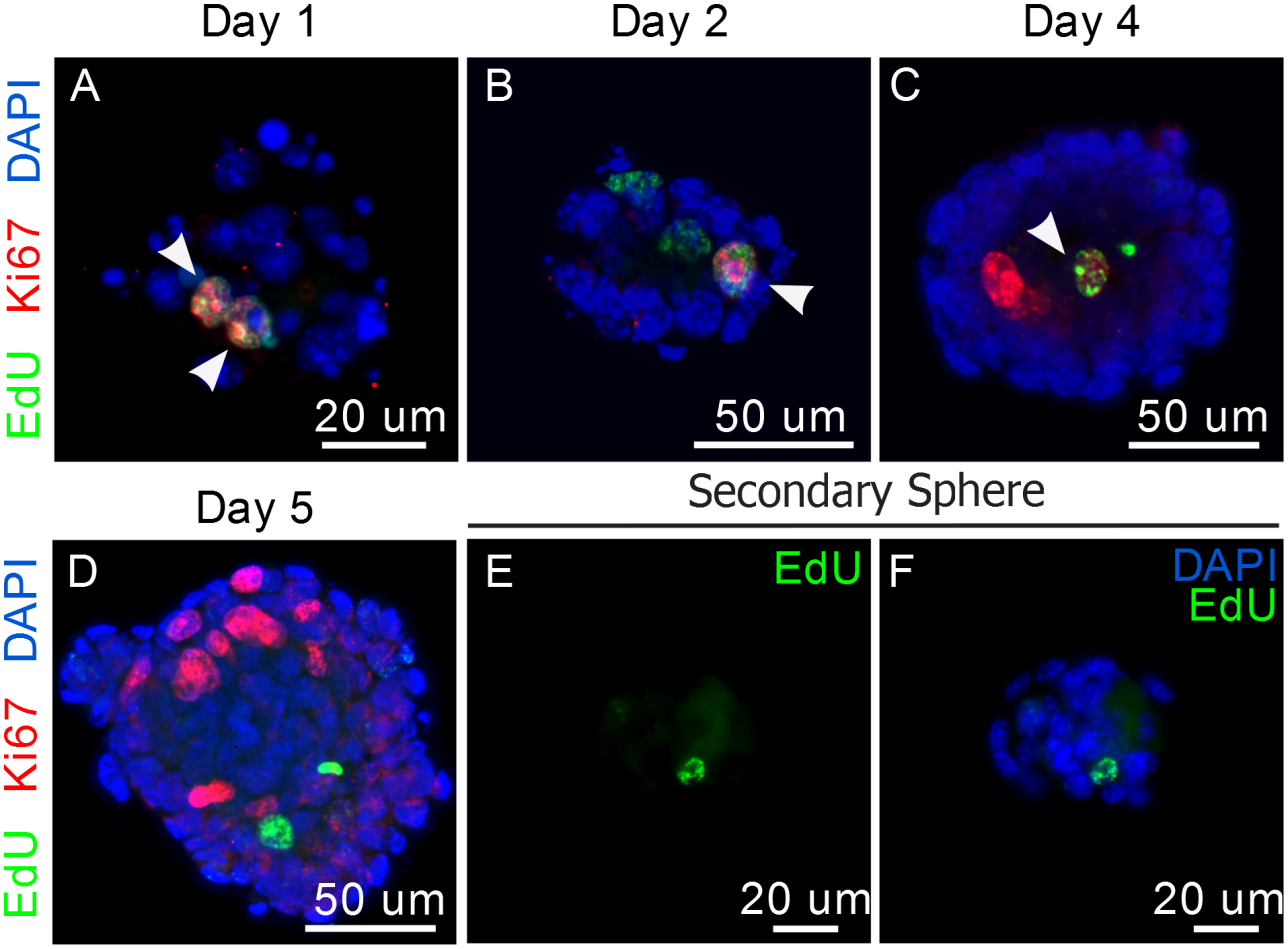
Proliferative potential of LRCs. A–C) Dual staining for EdU and Ki67 in parotid-derived spheres from 10-week old mice. Co-localization is shown with white arrowheads. D–F) Secondary spheres stained for EdU. G–I) Dual staining for EdU and Ki67 in parotid gland tissue sections of 10-week old mice. Zoomed region in yellow square is shown in I’. In both panels, Ki67 is shown in red, EdU in green, and DAPI in blue.

Spheres from parotid and submandibular glands were cultured as explained in the methods section. Spheres were first detected at day 2 after culture (Fig. 3A, B Day 2) and reached their maximum size around day 5 (Fig. 3). At day 5, lots of cells were detaching from the spheres (Fig. 3A, B Day 5) and cell death was greatly increased (data not shown). Strikingly, essentially every sphere contained at least one EdU LRC (Fig. 4A, D), and secondary spheres grown from dissociated primary spheres also contained EdU+ cells (Fig. 4E–F), suggesting that LRCs are involved in sphere formation.

To confirm LRCs have proliferative potential, we looked for co-localization of LRCs with the marker of proliferation Ki67 throughout sphere formation. Co-localization of EdU+ cells with Ki67 was observed during early stages of sphere formation (Figure 4A–C), confirming that LRCs have the potential to proliferate *in vitro*. At later time points in culture, non-LRCs, are highly proliferative, while LRCs seem to return to a state of quiescence (Fig. 4D).

### Salivary gland-derived spheres generate differentiated amylase-secreting cells

**Figure 5:**
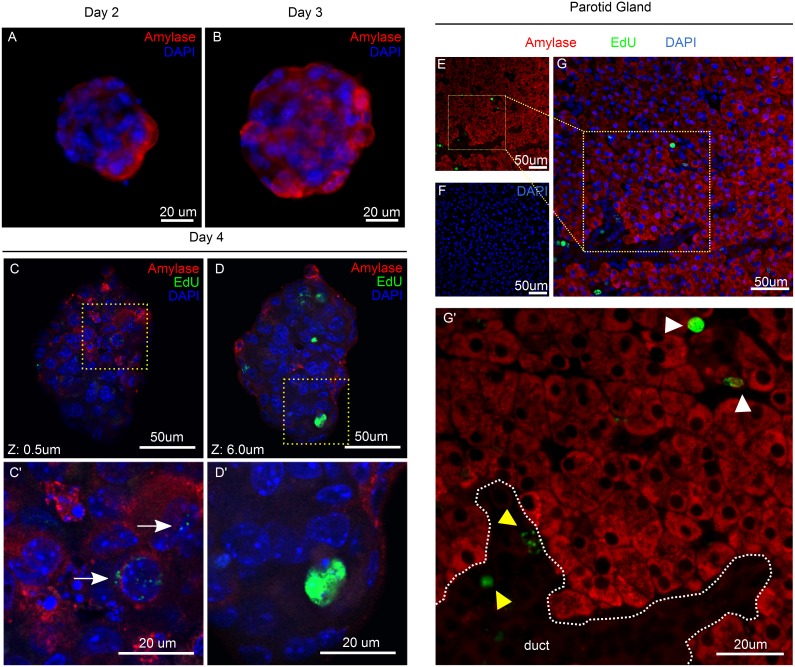
Differentiation of Salivary gland Spheres. A–B) Amylase staining (red) of parotid-derived spheres at days 2–3 in culture. C–D) Confocal images at Z = 0.5 um and Z = 6 um of double staining for amylase (red) and EdU (green) at day 4. Areas in yellow dashed squares are shown in C’ and D’. White arrow points at an amylase-positive cell with traces of EdU. Glands were obtained from mice at 10 weeks of age. E–G) Double immunofluorescence staining for Amylase (red) and EdU (Green) of parotid gland of 10-week old mice. White arrowhead points at LRCs in the acinar compartment; yellow arrowhead points at LRCs in ductal structures.

Because LRCs showed multiple features of progenitor cells, including the ability to expand and form spheres *in vitro*, we hypothesized that LRCs had the capacity to generate differentiated salivary gland cells. To evaluate the differentiation capacity of LRCs, we performed dual immunofluorescent staining of EdU and amylase on spheres derived from parotid gland from EdU-labeled mice. Since the beginning of sphere formation, amylase was detectable in only a few cells (Fig. 5A), but with longer periods in culture, there was an increase in the number of Amylase+ cells within the spheres (Fig. 5B–D). Importantly, LRCs did not co-localize with Amylase+ cells *in vivo* (Fig. 5G’) or *in vitro* (Fig. 5C–D), and only traces of EdU were detected in some Amylase+ cells within the spheres (Fig. 5C’, white arrow). This may represent some EdU+ cells that have proliferated early during culture (and therefore diluted the EdU label) and subsequently have undergone differentiation at later stages of culture. The loss of acinar cells is partly responsible for salivary gland dysfunction; therefore, the capacity to generate differentiated amylase-secreting cells has therapeutic potential.

### Salivary gland LRCs survive targeted radiation treatment to the head and neck

**Figure 6:**
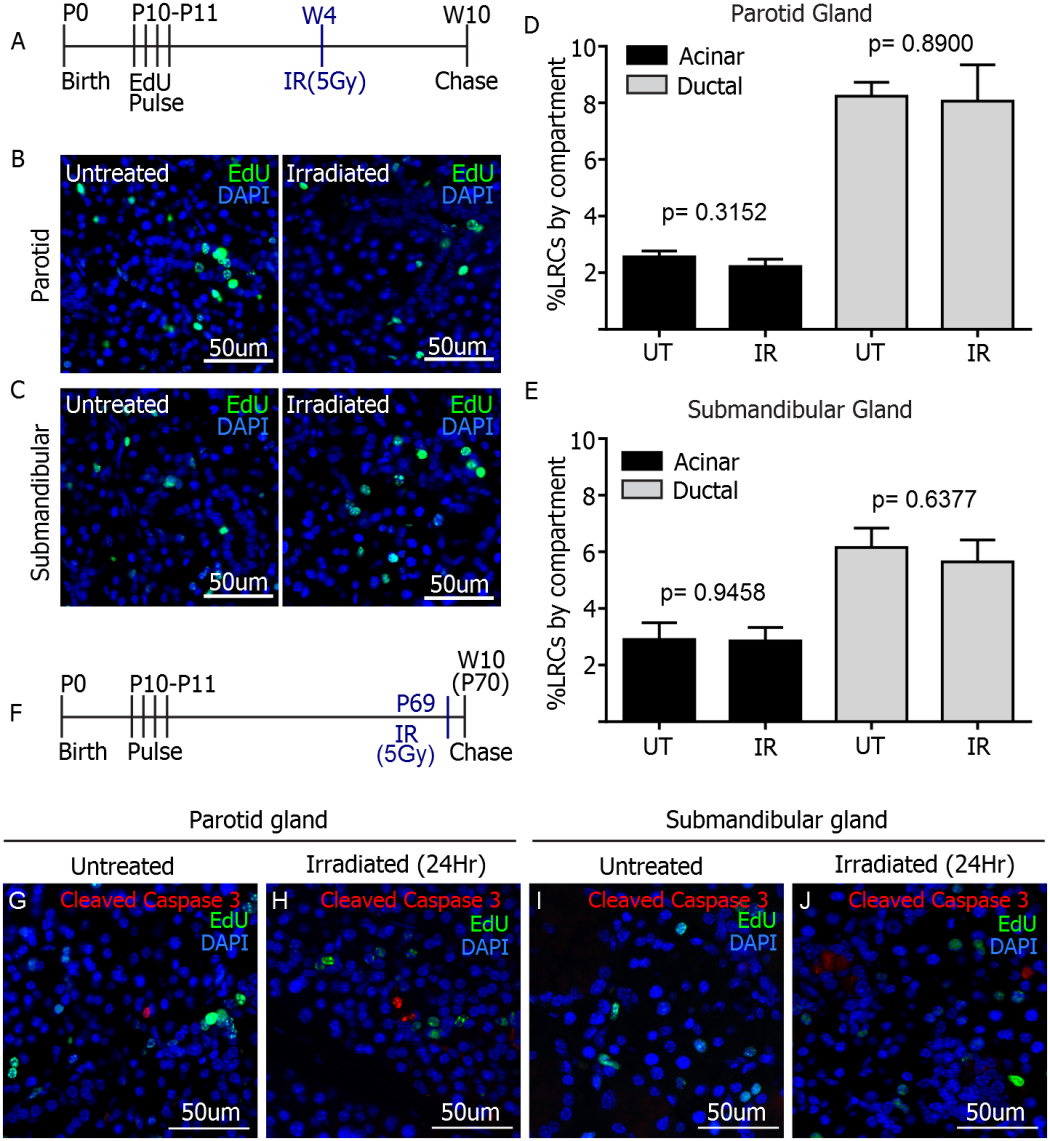
Effect of radiation on salivary gland LRCs. A) Experimental setup. A single 5 Gy dose of radiation was given at week 4 to EdU-pulsed animals (n = 7). Tissue was collected at week 10. Representative images of EdU staining of parotid (B) and submandibular (C) glands are shown for irradiated animals and untreated controls. EdU positive cells were quantified manually per individual compartment for both treatments and expressed as percentage of LRCs per compartment for both glands (D–E). P values were obtained with 2-sided unpaired t-test per compartment (n = 7 for irradiated groups, n = 12 for parotid untreated control group and n = 6 for submandibular untreated control group). F) Experimental setup. 5 Gy dose of radiation treatment was given 24-hours prior tissue collection to EdU-pulsed animals (n = 3). G–J) Immunofluorescence staining of Activated Caspase-3 (red) and EdU (green) in parotid and submandibular glands. No co-localization is observed.

As mentioned before, it has been postulated that radiation treatment causes loss and/or sterilization of salivary progenitor cells, impairing the ability of the tissue for self-repair [18]. Because LRCs comprised a mixed population of cells with progenitor capacity, we decided to evaluate the effect of targeted radiation treatment upon these populations. For this purpose, a group of EdU-labeled FVB mice, was subjected to a single 5 Gy dose of targeted radiation of the head and neck region at week 4 (Fig. 6A). A control group was labeled with EdU but was excluded from the radiation treatment for comparison. Mice were euthanized at week 10 and salivary glands were collected for analysis by immunofluorescence.

EdU staining of parotid and submandibular glands (Fig. 6B–C) revealed no differences in the percentage of LRCs present in either acinar or ductal compartments in irradiated samples when compared to untreated controls (Fig. 6D–E) (p>0.05 in all cases by unpaired 2-sided T-Test for 2 samples with equal variances, see radiation section of methods for number of samples). This particular finding indicated that the number of EdU+ progenitor cells was maintained long after radiation treatment. In addition, dual immunofluorescence staining of EdU and Cleaved Caspase 3 was performed on tissues collected 24 hours-post radiation treatment to evaluate apoptosis of LRCs at this particular time point. No co-localization between activated Caspase 3 and EdU was observed in either parotid or submandibular glands of 3 different mice (Fig. 6G–J). The preservation of LRCs following treatment, combined with their maintenance of proliferative potential *in vitro*, as well as the generation of amylase-secreting acinar cells within spheres, makes LRCs a valuable target for development of regenerative therapies, which could be applied to head and neck cancer patients undergoing salivary gland dysfunction as a consequence of radiotherapy.

## Discussion

Radiation-induced salivary gland dysfunction is the cause of miserable quality of life in the roughly 50,000 annual cases of head and neck cancer in the U.S [1], [3]. Here we present a model of label retaining cells in murine salivary glands, which can be used in the study of regenerative therapies for irradiated salivary glands. We demonstrate that label retaining cells (LRCs) encompass multiple populations of salivary progenitors, which have the ability to differentiate into amylase-secreting cells. Most importantly, we are the first group to demonstrate that the number of salivary gland progenitor cells is maintained long term following radiation, which makes them a valuable candidate for restorative therapies.

We were able to design a label retaining assay for the salivary glands in which we obtained comparable numbers of LRCs to those previously shown by Kimoto et al. [44]. Previous studies have used BrdU as a means to identify label retaining cells in several tissues, salivary glands included [32], [40], [44], [52], [53]. Currently, more flexible alternatives are available, such as EdU incorporation, which suppresses the use of antibodies and thus eliminates the problem of cross-reactivity with multiplexing staining [29], [34]. We chose postnatal day 10 as the pulse (labeling) time point based on a previous study of label retaining cells in the submandibular gland [44]. The rationale was that at P10, progenitor cells are still actively proliferating, allowing for incorporation of EdU; if cellular markers expressed at P10 are then found in co-localization with LRCs in adult salivary glands, it suggests that shortly after P10, these cells became and remained quiescent throughout the 8-week chase period, consistent with progenitor cell behavior.

We demonstrate here that LRCs are salivary gland progenitors, based on their co-localization with molecular markers associated with salivary progenitors and their maintenance of proliferative potential *in vitro*. Co-localization with c-kit was expected, since earlier studies demonstrated that c-kit+ cells were important progenitors during development [22], [26] and had partial regenerative potential upon transplantation to irradiated salivary glands [22]. However, it was surprising that co-localization between LRCs and c-kit was not observed in major ductal structures, where other progenitor markers such as K5 have been reported [21], [23]. This could likely signify that LRCs encompass a heterogeneous population of progenitor cells, distinctly localized to different compartments of the salivary glands. Indeed, we found Keratins 5 and 14 to be expressed in the major ducts of parotid and submandibular glands, but they rarely co-localized with LRCs (∼1%). Surprisingly, about 20% of the K5+ cells in the acinar compartment were LRCs. K5+ cells have been described as proximal progenitors during submandibular gland development [23], but in our study, the abundance of K5+ LRCs in the acinar compartment suggests that in the adult glands, a subset of K5+ cells might represent a population of progenitors restricted to a different lineage, possibly acinar or myoepithelial. K14 on the other hand, was highly expressed in ductal cells of the parotid gland, where *kit* mRNA was also expressed. Importantly, K14 has demonstrated a very important role in salivary gland organogenesis in combination with c-kit [26], and its co-localization with LRCs in a compartment where *kit* RNA is highly expressed, suggests that K14+ cells in adult parotid gland might also be important for gland homeostasis. Interestingly, similar to K5, K14 was found in the basal layer of major ducts, but didn’t co-localize with LRCs in that compartment. K14+ LRCs cells were found in small ducts of parotid, and sparsely localized in submandibular gland. These patterns of LRCs co-localization with the aforementioned markers support the existence of multiple progenitors within the salivary epithelium. In concordance with this, a study of murine sweat glands reported that LRCs comprised three populations of adult progenitors, all of which contribute to homeostasis of the glands [34].

In addition to the presence of progenitor markers in LRCs, their contribution to sphere formation further supports their role as adult progenitor cells. The sphere assay is based on the ability of stem/progenitor cells to proliferate, self-renew and differentiate in suspension [51], [54]. The sole presence of LRCs within the spheres at every time point, but most significantly, in secondary spheres, indicates that these are cells with high capacity to survive the *in vitro* environment in spite of lacking an extracellular matrix. Sphere assays, have been employed in a number of tissues, including pancreas [55], mammary glands [56], and neural tissue [54], [57], in which spheres are enriched with cells that harbor self-renewal and differentiation capacity.

Moreover, while salivary glands are known to hardly proliferate *in vivo* [58], LRCs became actively proliferating cells upon culture in sphere-forming conditions. A similar behavior has been observed in other models of label retaining cells in a variety of tissues [37], [40]–[42]. Importantly, proliferation of LRCs in these tissues was often related to damage repair and homeostasis. Oliver, J. et al. [41], [42] demonstrated that LRCs in the renal papilla were directly involved in renal repair upon a transient ischemic event. Similar to our study, LRCs in the papilla were essentially quiescent in homeostasis but showed extensive proliferative potential otherwise. In their study, proliferation was triggered by tissue injury, whereas in our model it was activated upon culture *in vitro*. One more study in lacrimal glands [40], also showed proliferation of lacrimal LRCs, which was initiated by induction of a severe inflammatory response. It is also noteworthy that salivary gland LRCs did not show signs of amylase expression both *in vivo* and *in vitro*, suggesting an undifferentiated phenotype.

The most important finding in the present study is that the percentage of salivary gland LRCs in the submandibular and parotid salivary glands is maintained after radiation. It has been previously demonstrated that levels of apoptosis in the parotid gland peak at 24 hours following radiation [45]–[48]; therefore the level of apoptotic LRCs was evaluated at this time point. We observed that LRCs do not undergo apoptosis at this particular time after radiation in both parotid and submandibular glands. However, due to rapid clearance of apoptotic cells *in vivo*, the possibility remains that some LRCs could undergo apoptosis prior to the analyzed time point. It has been speculated that radiation therapy kills, or sterilizes the residing progenitors of the salivary glands [18], but this was never addressed experimentally. Transplantation assays have shown that c-kit+ progenitors have potential to partially restore salivary gland function; however, it is unclear whether the endogenous c-kit+ cells of the irradiated gland respond in any way to radiation treatment. In contrast, a more recent study [27] reported that K5+ progenitors survived in explants of murine embryonic SMG after *ex vivo* radiation treatment and retained the ability to regenerate the gland upon administration of Neurturin. The same study found that K5+ cells remained after radiation in human biopsies from adult salivary glands, and concluded that maintenance of parasympathetic innervations after radiation could aid in regeneration of glandular function. An explanation for maintenance of K5+ progenitors after radiation is that they are in fact resistant to radiation and escape apoptosis, or that the loss of endogenous K5+ cells triggers a proliferative response in the surviving progenitors, maintaining a constant number. Whether these observations hold true for progenitors of the parotid gland remains to be elucidated. Importantly, however, LRCs are maintained in both submandibular and parotid salivary glands after radiation (Fig. 6D–E), and similar to K5+ progenitors, there could be potential for regeneration upon administration of the right stimuli.

In contrast with our study, it was very recently reported that Lgr5+ LRC stem cells underwent apoptosis due to extensive radiation-induced DNA damage, but the surviving LRCs were able to restore function [37]. This emphasizes the importance of understanding the mechanisms of radiation damage to tissue-specific stem cells. Thus, it is relevant to further study how radiation therapy specifically affects salivary gland LRCs, to facilitate the development of regenerative therapies for patients undergoing radiation-induced xerostomia.

## Supporting Information

Figure S1 A–C) FACS analysis of 2 Edu-labeled mice (B–C) and an unlabeled control (A). P2 is the population of EdU+ sorted cells. D–E) Microscope images from non-sorted cells from unlabeled control. F–K) Microscope images of sorted EdU+ cells from labeled mice. L) RNA analysis comparing gene expression of EdU+ (P2) sorted cells versus EdU− (P3) sorted cells.
(TIF)

Figure S2 Full size representative images of immunofluorescence staining for Keratin 14 (A–D), Keratin 5 (E–L), and Smooth Muscle alpha Actin (M–P) in both glands from 10-day old and 10-week old animals. Q–T) Images of FISH for kit mRNA in both glands from 10-day old and 10-week old animals. Yellow squares in all images indicate the corresponding zoomed-in areas shown in Figure 2.
(TIF)
