# Supplementary figures and images for: Label-Retaining Cells in the Adult Murine Salivary Glands Possess Characteristics of Adult Progenitor Cells
Source: PLoS One. 2014 Sep 19;9(9):e107893. doi: 10.1371/journal.pone.0107893 (PMC4169596; doi:10.1371/journal.pone.0107893)

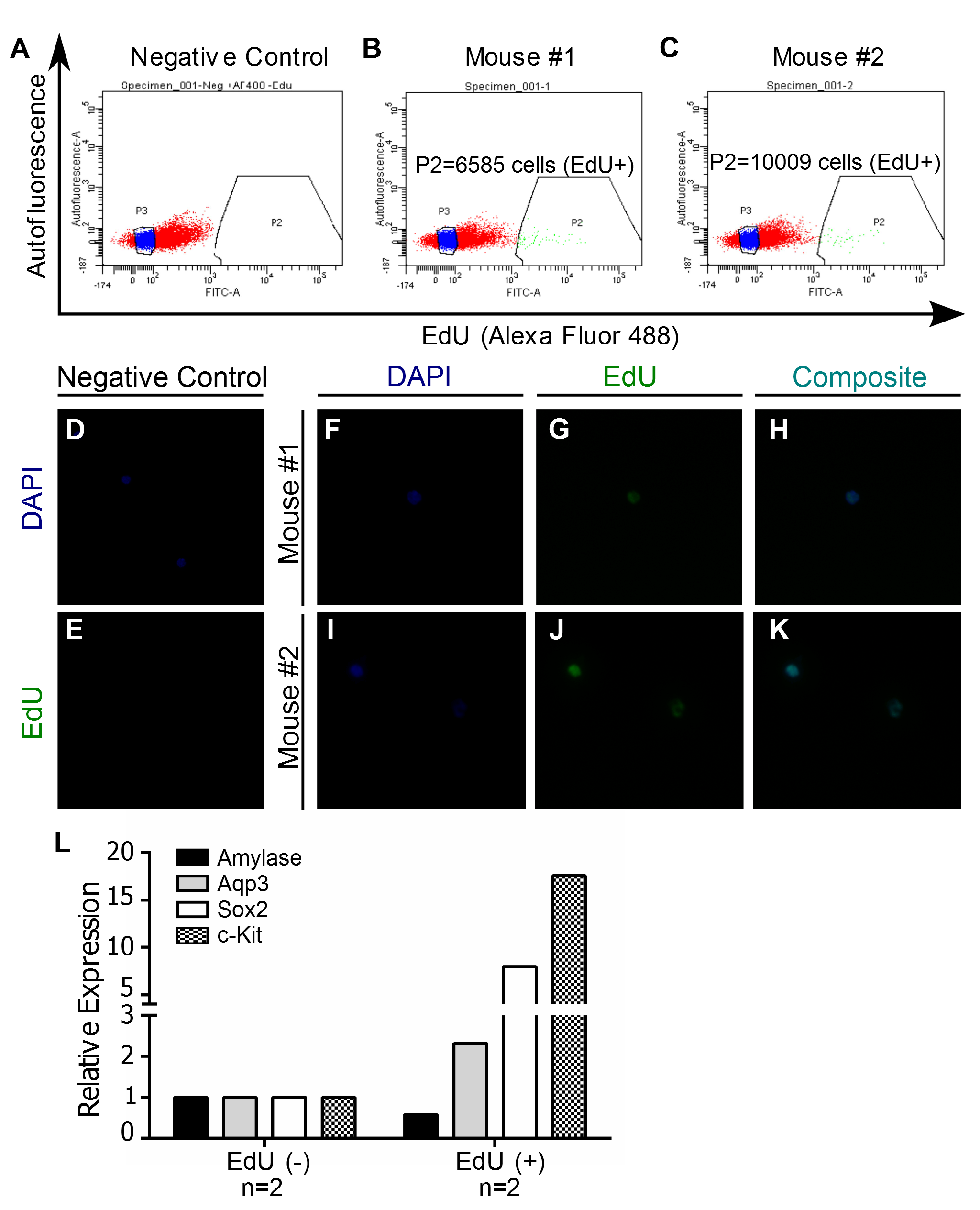

Supplement: Figure S1 — A–C) FACS analysis of 2 Edu-labeled mice (B–C) and an unlabeled control (A). P2 is the population of EdU+ sorted cells. D–E) Microscope images from non-sorted cells from unlabeled control. F–K) Microscope images of sorted EdU+ cells from labeled mice. L) RNA analysis comparing gene expression of EdU+ (P2) sorted cells versus EdU− (P3) sorted cells. (TIF) [file pone.0107893.s001.tif]

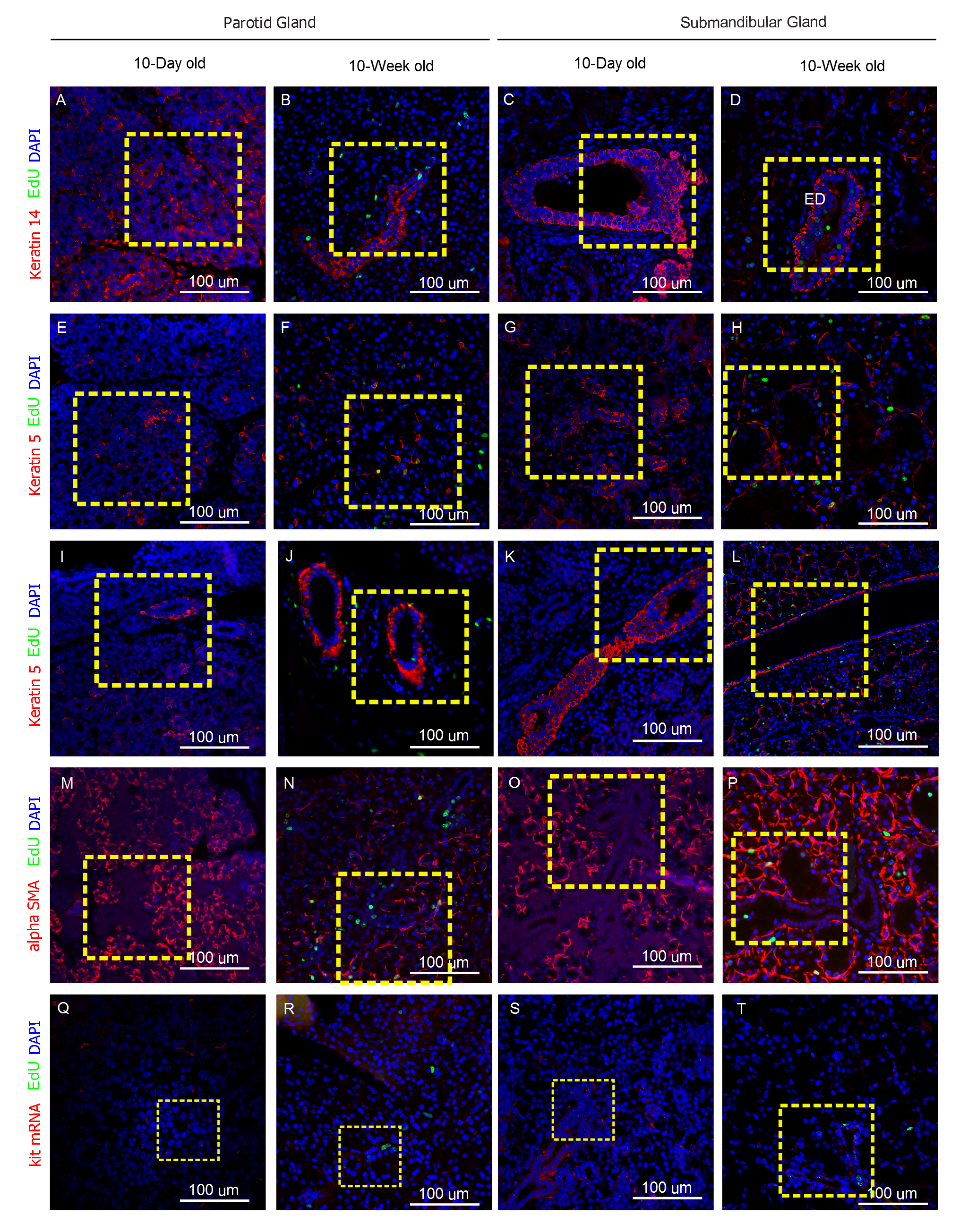

Supplement: Figure S2 — Full size representative images of immunofluorescence staining for Keratin 14 (A–D), Keratin 5 (E–L), and Smooth Muscle alpha Actin (M–P) in both glands from 10-day old and 10-week old animals. Q–T) Images of FISH for kit mRNA in both glands from 10-day old and 10-week old animals. Yellow squares in all images indicate the corresponding zoomed-in areas shown in Figure 2. (TIF) [file pone.0107893.s002.tif]
